# Effects of Displacement Piles for Dike Reinforcement on Adjacent Buildings

**DOI:** 10.1007/s10706-025-03438-y

**Published:** 2025-09-22

**Authors:** Majd Ahmad, Ronald B. J. Brinkgreve, Sebastiaan N. Jonkman

**Affiliations:** 1 Faculty of Civil Engineering and Geosciences, Department of Hydraulic Engineering, Delft University of Technology, Delft, The Netherlands; 2 Faculty of Civil Engineering and Geosciences, Geo-Engineering Section, Delft University of Technology, Delft, The Netherlands

**Keywords:** Displacement piles, Dike reinforcement, Finite element method, Soil-structure interaction, Installation-induced displacements

## Abstract

This study presents a comprehensive numerical investigation into the use of displacement piles as a reinforcement measure for river dikes founded on soft soil, with a particular focus on geotechnical performance, macro stability, and impacts on nearby buildings. A finite element model is developed using parameters derived from a representative Dutch dike case (Bergambacht), incorporating the Hardening Soil, Soft Soil Creep and NGI-ADP-SHANSEP models to capture soil behaviour. Pile installation is simulated through the application of lateral volumetric strain, with varying pile diameters, spacings, and locations within the dike profile. The equivalent diameters used in the analysis range from 10 to 40 cm, corresponding to pile walls with diameters between 25.5 and 100 cm when the spacing equals the diameter. The pile wall location varies from the dike toe up to 21 m away, which is at the outer crest, with a varied length reaching -12 m NAP. A two-storey building on deep pile foundations is included to assess the effect of installation-induced displacements, with its location ranging from 5 to 20 m from the dike toe. Results show that positioning the pile wall within the inner slope offers the best balance between increased factor of safety, reduced required pile length, and acceptable levels of deformation. However, the installation process can generate significant horizontal displacements, particularly near the dike toe, which may compromise adjacent structures. The study finds that displacement piles are unsuitable within 10–15 m of existing buildings unless smaller pile diameters or alternative installation methods are used. Soil stiffness and installation-induced stresses also play a key role, highlighting the importance of site-specific assessments and careful design calibration using field data.

## Introduction

In the Netherlands, around 1,500 kms of flood defences are expected to require reinforcement by 2050 (National Delta Programme 2023). These defences, which include both dikes and dunes, are essential for safeguarding the country’s low-lying regions from flooding. As the primary flood protection structures, dikes must comply with strict safety standards. Climate change is causing an increase in sea level and river discharge (Hermans, et al. 2025), which necessitates the heightening of dikes to protect the polders from overtopping or overflowing. However, increasing the height of a dike can reduce its overall stability, particularly with respect to macro-stability, unless it is properly reinforced. This could result in the dike no longer meeting safety requirements, posing a risk of severe societal and economic consequences. In addition, safety standards for Dutch flood defences have been revised recently and have become stricter, also necessitating additional reinforcements.

Traditionally, stabilizing a dike after increasing its height involves widening it, often by adding berms. However, this method can be impractical in densely built-up areas where existing structures limit the space available for widening. In such cases, alternative reinforcement methods are used to allow for dike stabilization while preserving the existing buildings in the area. These include embedded retaining elements such as sheet piles (both anchored and unanchored) (Gallala and Kullolli 2025), diaphragm walls, soil mix walls, cofferdams, and bored piles walls (Larsen, Lubking and Breedeveld 2013), (Zhang, Xie and Peng 2024). Geosynthetic-reinforced embankments (Wu, et al. 2022) are widely used around the world, but they are not commonly applied to dike reinforcement in the Netherlands. These solutions aim to enhance the stability of the dike while minimizing the impact on surrounding infrastructure.

Due to the complexity of soil-structure interactions involved in dike reinforcement, analytical methods are insufficient for design purposes. Instead, the Finite Element Method (FEM) is commonly employed to model and analyse these interactions accurately (Lengkeek 2022), (Zhao, et al. 2025). The guidelines provided by the Dutch national program for reinforcement of flood defences (Larsen, et al. 2020) help ensure that any longitudinal reinforcement structure meets the required reliability standards for macro stability. Despite conservative design practices, excessive deformation near the dike toe has been observed in some projects, occasionally resulting in damage to the very buildings that the approach sought to preserve.

Among the available reinforcement methods, (semi-)continuous rows of piles in the dike’s longitudinal direction have increasingly been used (Iqbal and Tanaka 2024). There are two main categories of pile installation: displacement and non-displacement methods. Displacement piles are driven, jacked, or vibrated into the ground without removing soil, causing the surrounding soil to deform or compact (Shao, et al. 2025). While this densification can improve strength, it may also lead to horizontal ground movements and pose a risk to adjacent structures due to lateral soil displacement (van Overstrat en Kruijss 2019).

Non-displacement piles, on the other hand, are installed by first removing soil to form a borehole, which is then filled with concrete or other materials. This method requires casing or stabilizing fluids, such as bentonite, to maintain the stability of the borehole during construction. Although this technique minimizes lateral ground movement, it can lead to settlement due to soil removal (Ljungberg and Aström 2025). In sensitive areas, like dikes, this technique is generally not recommended, as removing soil can potentially affect dike stability. Therefore, such interventions near or within dikes should be approached with caution.

Past projects highlight the implications of each approach. In the 2011 Bergambacht–Schoonhoven (BAS) dike reinforcement (Arcadis 2003), displacement piles installed at the dike crest caused soil deformation and damage to nearby buildings, prompting a switch to a deep wall alternative (POVM 2020). Conversely, in the Kinderdijk–Schoonhovense Veer (KIS) project (De Bruijn and Fransen 2024), a soil-removing drilling method was used, involving a bucket auger and temporary casing to mitigate ground disturbance. Nevertheless, this method also presented issues following the completion of the dike reinforcement, including water infiltration that led to wet spots within the dike body and near houses, as well as damage to adjacent buildings (Waterschap Riviereland 2024).

The guidelines lack clear recommendations on the minimum distance required between existing buildings and the dike toe to avoid damage caused by the installation of displacement piles. It is also a knowledge gap that the guidelines do not specifically indicate how to assess the effects of piles on nearby buildings. In addition, some specific effects such as the displacement of soil around the pile and vibration during installation remain unexplored.

Therefore, this study examines the impact on nearby structures of using displacement piles for dike reinforcement, both positioned on the slope and at the crest. The research aims to identify optimal pile configurations and installation strategies that enhance dike stability while minimizing structural risks to adjacent infrastructure in densely built environments.

This study contributes a novel approach to dike reinforcement by integrating geotechnical, structural and soil-structure interaction modelling to assess the impact of displacement pile installation on adjacent buildings, an aspect largely overlooked in current design guidelines. By simulating pile-induced soil displacement using lateral volumetric strain within finite element models in Plaxis 2D, the research offers a practical framework for evaluating deformation risks in built-up areas. The comparative analysis of structural modelling techniques and sensitivity to soil stiffness further enhances the applicability of the findings to real-world reinforcement projects.

The remainder of this paper is organized as follows. Section 2 describes the schematization of the finite element model used in this study, including the geometry, phreatic lines, soil models and material properties. Section 3 outlines the analysis steps used to calculate the factor of safety and deformations for a dike reinforced with a pile wall. Section 4 presents a method for investigating the effect of displacement pile wall installation on adjacent buildings. Finally, Sect. 5 provides the results and discussion, followed by the conclusion.

## Schematization and Soil Properties

**Fig. 1:**
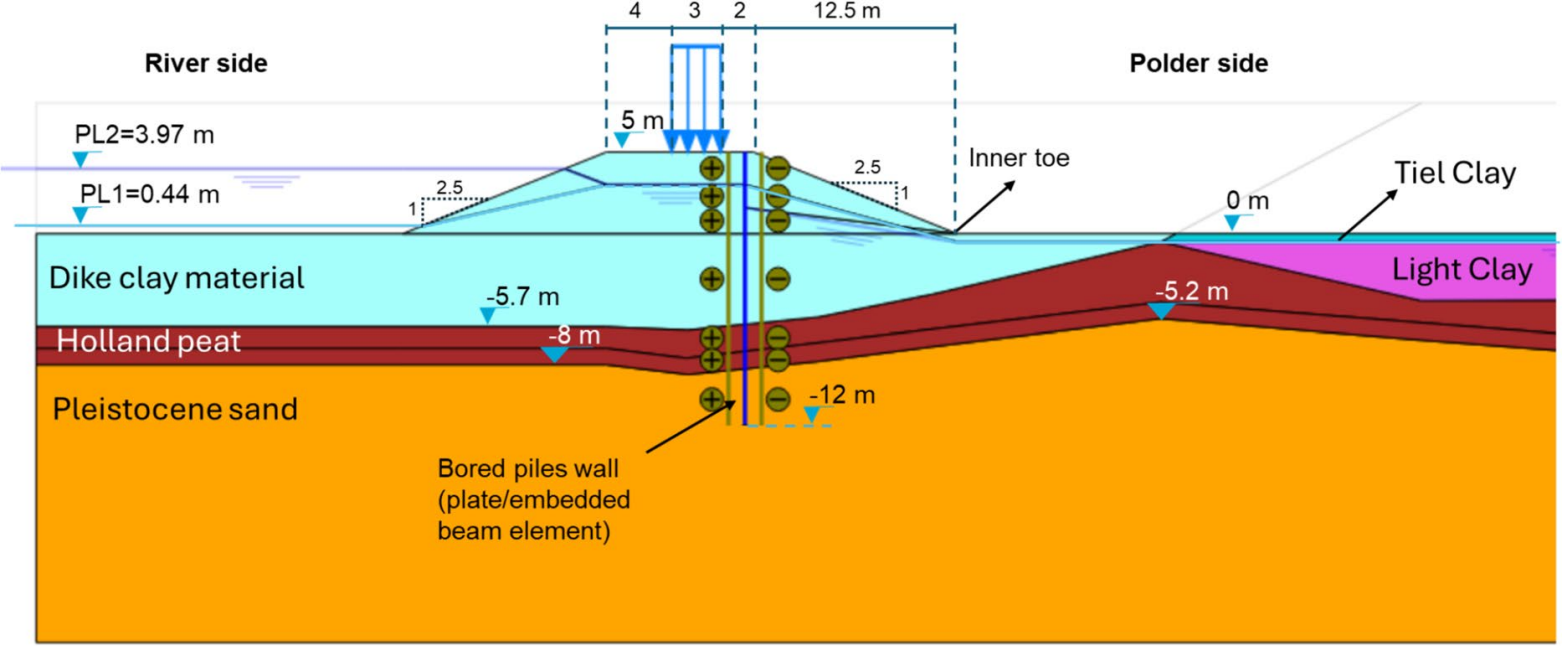
Schematization of the finite element model for the Bergambacht case

The schematization of the FEM model in Plaxis 2D (Fig. 1) is based on the location of the old Lekdijk near Bergambacht, where a shear test was conducted in 2001 (Lindenberg, et al. 2002). This site was chosen due to the availability of extensive measurements and detailed local soil investigations. As a result, it was also used as an example in the guidelines (POVM 2020) to illustrate design calculations for various reinforcement techniques. In this study, the same case is further utilized to examine the impact of displacement piles on nearby buildings.

The Hardening Soil model (HS) was used for the Pleistocene sand layer, while the Soft Soil Creep (SSC) and the NGI-ADP-SHANSEP models were used for clay and peat soils. The parameters are summarized in (Table 1, 2 and 3). The SSC model (Vermeer, Stolle and Bonnier 1998), (Brinkgreve 2004) is used with a drained behaviour and low characteristic values for shear strength properties to determine the initial stress state under high water conditions. Furthermore, it takes into account the effect of the soft soil long term settlement on the normal forces applied to the wall (negative skin friction/adhesion) in a 100-years creep phase. Before conducting the safety analysis (shear strength reduction method), the development of the over-consolidation ratio and the stress conditions reached after applying the high-water level are vital to calculate the shear strength in the NGI-ADP model based on SHANSEP formulation.

**Table 1:** Soft Soil Creep model properties

Parameter	Name	Dike clay	Holland peat	Light clay	Tiel clay	Gorkum clay	Unit
Saturated unit weight	${\gamma }_{sat}$	18.45	10.3	11.86	14.23	13.88	kN/m^3^
Modified compression index	${\lambda }^{*}$	0.0654	0.247	0.178	0.127	0.2176	–
Modified swelling index	${\kappa }^{*}$	0.0077	0.0307	0.0378	0.0204	0.0216	–
Modified creep index	${\mu }^{*}$	0.0039	0.0164	0.0119	0.0094	0.0107	–
Poisson ratio	${\nu }_{ur}$	0.2	0.15	0.15	0.25	0.15	–
Cohesion	${c}{\prime}$	2	1	1	1	1	kN/m^2^
Friction angle	${\phi }{\prime}$	27.3	29.78	31.23	18.9	35.6	^o^
Dilatancy angle	${\psi }{\prime}$	0	0	0	0	0	^o^
Interface reduction factor	${R}_{int}$	0.73	0.67	0.67	0.67	0.67	–
K_0_ determination	*K*_*0*_^*nc*^	0.4	0.34	0.64	0.6	0.3	–
Pre-Overburden pressure	POP	30	10	10	27	10	kN/m^2^

**Table 2:** Hardening Soil model properties

Parameter	Name	Pleistocene Sand	Unit
Saturated unit weight	${\gamma }_{sat}$	20	kN/m^3^
Poisson ratio	${\nu }_{ur}$	0.2	–
Secant stiffness (triaxial)	${E}_{50}$	35,000	kN/m^2^
Tangent stiffness (oedometer)	${E}_{Oed}$	35,000	kN/m^2^
Unloading/reloading stiffness	${E}_{ur}$	100,000	kN/m^2^
Stress dependency power	m	0.5	–
Failure ratio	R_f_	0.9	
Cohesion	${c}{\prime}$	0.1	kN/m^2^
Friction angle	${\phi }{\prime}$	32.5	^o^
Dilatancy angle	${\psi }{\prime}$	0	^o^
Interface reduction factor	${R}_{int}$	0.62	–
K_0_ determination	*K*_*0*_^*nc*^	0.426	–
Over-consolidation ratio	OCR	1.1	–

**Table 3:** NGI-ADP-SHANSEP model properties

Parameter	Dike Clay	Holland Peat	Light Clay	Tiel Clay	Unit
${{\varvec{\gamma}}}_{{\varvec{s}}{\varvec{a}}{\varvec{t}}}$	18.45	10.35	11.86	14.23	kN/m^3^
${\varvec{G}}/{{{\varvec{S}}}_{{\varvec{u}}}}^{{\varvec{A}}}$	193	24	26.13	25.57	–
${{{\varvec{\gamma}}}_{{\varvec{f}}}}^{{\varvec{C}}}$	10.8	12.07	9.66	9.74	%
${{{\varvec{\gamma}}}_{{\varvec{f}}}}^{{\varvec{E}}}$	15.12	16.9	14.49	14.6	%
${{{\varvec{\gamma}}}_{{\varvec{f}}}}^{{\varvec{D}}{\varvec{S}}{\varvec{S}}}$	12.6	14.49	12.07	12.17	%
${{{\varvec{S}}}_{{\varvec{u}}}}^{{\varvec{P}}}/{{{\varvec{S}}}_{{\varvec{u}}}}^{{\varvec{A}}}$	0.98	0.98	0.98	0.98	–
${{\varvec{\tau}}}_{0}/{{{\varvec{S}}}_{{\varvec{u}}}}^{{\varvec{A}}}$	0.5	0.5	0.5	0.5	–
${{{\varvec{S}}}_{{\varvec{u}}}}^{{\varvec{D}}{\varvec{S}}{\varvec{S}}}/{{{\varvec{S}}}_{{\varvec{u}}}}^{{\varvec{A}}}$	0.99	0.99	0.99	0.99	–
${\varvec{\nu}}$	0.2	0.15	0.15	0.25	–
${{\varvec{\nu}}}_{{\varvec{u}}}$	0.495	0.495	0.495	0.495	–
$\boldsymbol{\alpha }$	0.25	0.29	0.25	0.18	–
**Power (*m*)**	0.76	0.76	0.76	0.76	–
${{\varvec{S}}}_{{\varvec{u}},{\varvec{m}}{\varvec{i}}{\varvec{n}}}$	5	5	5	5	kN/m^2^

The NGI-ADP is a 'total stress' model (Grimstad et al. 2010), (Zhang, et al. 2024b). The abbreviation ADP stands for Active, Direct Simple Shear and Passive. This means that the model takes into account possible different values of the undrained shear strength in the active ${{(S}_{u}}^{A})$, neutral (direct simple shear, ${{S}_{u}}^{DSS}$) and passive ${{S}_{u}}^{P}$ part of the sliding plane, in combination with the different levels of plastic shear strain at which the peak values for the shear strength are reached (${{\gamma }_{f}}^{C},{{\gamma }_{f}}^{E},{{\gamma }_{f}}^{\text{DSS}}$ for compression, extension and direct simple shear, respectively). However, within the guidelines, no distinction is made between active and passive shear strength. The ratios should therefore generally be chosen close to 1, except in a possible uplift zone. The ratio between the unloading/reloading shear modulus (*G*) over the active shear strength is also determined, in addition to the initial mobilization ${\tau }_{0}$ and the drained and undrained Poisson ratio $\nu$ and ${\nu }_{u}$.

With each switch from HS or SSC to the NGI-ADP- SHANSEP model (Panagoulias and Brinkgreve 2017), PLAXIS determines the undrained strength associated with the largest effective principal stress ${\sigma }_{1}{\prime}$ at that time, in combination with the current pre-consolidation stress value ${\sigma }_{1,max}{\prime}$.

1 $${S}_{u}={\sigma }_{1}{\prime}\alpha {\left(\frac{{\sigma }_{1,\text{max}}{\prime}}{{\sigma }_{1}{\prime}}\right)}^{m}={\sigma }_{1}{\prime}\alpha {\left(OCR\right)}^{m}$$

In which $\alpha$ and *m* are normalized soil parameters, and *OCR* is the Over-Consolidation Ratio.

Based on surface water measurements, the average water level (PL1) is taken at NAP + 0.44 m. The extreme water level (PL2) is determined based on hydrological study including the expected rise of sea level and a margin of safety. The high-water level is taken at NAP + 3.97 m. PL1 and PL2 are shown in Fig. 1. The pressure in the aquifer at the daily conditions (PL3) and extreme condition (PL4) were determined based on (TAW 2004) for the case of clay dike on soft subsurface. The bottom 1 m of the soft soil was taken as the penetration layer. In the absence of information about permeability differences, linear interpolation of pore pressure is used between the top of the penetration layer and the 'layer separation' along the phreatic line.

Two different types of models were developed to assess the effects of piles. In Case I, the factor of safety (FOS) and deformations are calculated following the Dutch guidelines (POVM 2020). This model employs a semi-probabilistic approach to meet the permissible probability of failure using partial safety factors. The aim of this case is to investigate the effect of pile wall location on the factor of safety and to compare two finite element modelling approaches for piles: plates and embedded beams. Case II examines the effect of installing displacement piles in the dike on adjacent buildings by applying a horizontal volumetric strain (εₓₓ) to a thin concrete column. In both cases, the location of the bored pile wall in the dike is varied starting from the inner toe of the dike up to a distance of 21 m (close to the outer crest).

## Factor of Safety and Deformations Calculations Based on the Guidelines (Case I)

For (FOS) calculations, the bored piles wall is simulated with a plate element in a 2D plane strain model. In a discontinuous wall construction with openings, the structural elements are subjected to soil loads that extend beyond the width of the individual elements. As long as the openings remain limited, a 2D analysis can incorporate an equivalent bending stiffness (*EI*_*eq*_) and an equivalent axial stiffness (*EA*_*eq*_) per meter of wall. For bored piles, the shell factor (*S)* must be used as indicated in the design guideline for piles horizontally loaded by the ground (CUR 2010). The shell factor is the ratio of the earth pressure on the pile (with diameter *D*) and the earth pressure on the wall calculated in 2D. The following applies to the equivalent bending stiffness of the wall:

2 $${\text{EI}}_{\text{eq}}\text{ = }\frac{{\text{EI}}_{\text{pile}}}{\text{S.D}}$$

The shell factor is a factor by which a 2D method, which is used to calculate a pile horizontally loaded by soil, is adjusted in such a way that the result of this method is an approximation of the actual pile load and pile displacement (Begemann and de Leeuw 1972), (Hoefsloot 2009).

The shell factor accounts for several important aspects:

- The wedge of soil mobilized during loading is wider than the physical width of the pile.
- For a pile subjected to horizontal soil loading, shear stresses can develop along the sides of the pile, as the soil has the ability to flow around it. In contrast, such shear stresses do not occur in the case of a wall subjected to similar loading conditions.
- In three-dimensional conditions, failure planes differ from those predicted by 2D modelling.
- The passive soil pressure behind a pile differs from that behind a wall. For a pile, the rear soil pressure tends toward zero, and a gap may even form due to the ability of soil to flow around the pile. For a wall, significant passive pressures develop at the rear face.

In order for the structural elements to act as a wall and the 2D FEM analysis to be sufficient for the cutting test, the centre-to-centre distance should not exceed two times the diameter of the pile with maximum spacing of 1 m. In this case, the piles have a diameter of 0.3 m, and the centre-to-centre distance is 0.6 m. The elasticity modulus of the concrete pile is *E* = 35 GPa and the shell factor for clay from CUR288 is 1.4–1.8. The average of *S* = 1.6 is used here.

An accurate determination of the pile wall stiffness would require the use of *M–κ* diagrams, which account for the non-linear bending behaviour of concrete walls ( Huang, et al. 2025). These diagrams depend, among other factors, on the quality of the concrete and reinforcement, the effective concrete cross-section, axial forces, and the position and dimensions of the steel reinforcement. However, the structural design of the pile elements is beyond the scope of this paper, and assuming an elastic plate element is sufficient for the purposes of this study. The properties of the plate element are presented in Table 4.

**Table 4:** Material properties of the pile wall (plate properties)

Parameter	**Material type**	**Weight**	**Isotropic**	**Axial stiffness1**	**Bending stiffness**	**Poisson ratio**
symbol	–	w	–	*EA*_*1*_	*EI*	*ν*
Value	Elastic	7	No	$20.2*{10}^{6}$	$29*{10}^{3}$	0
Unit	–	kN/m/m	–	kN/m	kNm^2^/m	–

The interaction between soil and structure is modelled using interface elements, which represent deformable connections between the ground and the structure in both sliding and normal directions. Shear strength within these elements is typically represented using the Mohr–Coulomb criterion. For models such as HS and SSC, the mobilized friction angle or undrained shear strength is derived from the properties of the adjacent soil elements. This value is then reduced by a reduction factor, ${R}_{int}$, which reflects the relative roughness between the structural surface and the soil grain size. However, in the case of the SHANSEP NGI-ADP model, the strength and stiffness parameters of the interface elements must be specified manually.

To account for the effects of soil subsidence on structural forces, a creep phase of 100 years (the expected lifespan of the structure) can be included in the analysis. This should be combined with a point spring with high stiffness at the pile toe to limit the vertical movement of the pile. Settlement induces negative skin friction (or adhesion in fine soil) on walls or piles, which increases the axial (normal) force. This effect is typically critical for steel structures.

For concrete structures, however, a low axial force is often more critical, as it increases the likelihood of flexural cracking. Therefore, to conservatively estimate the normal stress in concrete walls (i.e., to ensure it remains low), the creep phase should be omitted, and point resistance should not be applied. Plaxis also allows for the use of embedded beam elements, which consist of beam components with embedded interface elements to model the interaction between the pile and surrounding soil, both along the pile shaft and at the pile toe (Sluis 2012), (Granitzer, et al. 2024). These elements are typically used to simulate structural components with spacing, such as piles, grouted anchors, and rock bolts (Plaxis reference manual).

This study presents a comparison of the resulting factors of safety and displacements when using embedded beam elements versus conventional plate elements with stiffness reduction based on shell factor (*S)*. The material properties used for the embedded beams are provided in Table 5.

**Table 5:** Material properties of the pile wall (embedded beams)

Parameter	Name	Pile wall	Unit
Material type	–	Elastic	–
Unit weight	$\gamma$	7	kN/m^3^
Pile spacing	*L*_*spacing*_	0.6	m
Cross section type	–	Predefined	–
Pre-defined cross section type	–	Solid circular beam	–
Diameter	–	0.3	–
Stiffness	*E*	$35*{10}^{6}$	kN/m^2^
Axial skin resistance	*Axial skin resistance*	Layer dependant	–
	*T*_*max*_	500	kN/m
Lateral resistance	–	Unlimited	
Base resistance	*F*_*max*_	400	kN

The calculation phases follow the steps described in (POVM 2020).

- Steps to reach the initial state of the dike before reinforcement.
- The initial phase models the existing dike and generates the initial stresses using the K₀ procedure, while accounting for the soil’s loading history through the Pre-Overburden Pressure (POP). Since the stresses are not fully in equilibrium at the end of this phase, the imbalance is corrected in the subsequent step.
- Stresses are redistributed and their directions are adjusted to achieve equilibrium in the plastic-nil phase, during which no additional loads are applied.
- The subsidence of the soft soil prior to the application of reinforcement is accounted for in this phase. A 10-year period is assigned in the plastic calculation to accumulate creep strains in the SSC model.
- The reinforcement is activated along with the interface elements.
- Apply extreme water level and activate distributed load of 13 kN/m with 3 m width.
- SSC model is switched to the NGI-ADP-SHANSEP model with undrained behaviour.
- Switch to SHANSEP without applying factors of safety to check for deformations (Serviceability limit state).
- Apply damage factor (${\gamma }_{d})$ and model factor (${\gamma }_{n})$ to check for structural forces.
- Global safety factor is calculated using the strength reduction method (${\gamma }_{d}$ and ${\gamma }_{n}$ are applied).

The damage factor ${\gamma }_{n}$ and model factor ${\gamma }_{d}$ are calculated to satisfy a maximum permissible probability of flooding of 1/3000 per year. The partial factors of safety are applied to the characteristic soil strength in the FEM calculation. For the structural check, a factor ${\gamma }_{n}.{\gamma }_{d}=1.06*1.16=1.23$ is applied to the soil strength in total. Hence, a reduction factor of 1.23 is applied to cohesion (c`), the tangent of angle of friction (${\varphi }{\prime}$) and undrained shear strength (*S*_*u*_) in phase 4b before conducting the structural checks and the safety analysis. The final factor of safety resulted from the *c*, $\varphi$ reduction calculation is compared to the schematization factor ${\gamma }_{b,GEO}=1.1$. Therefore, the factor of safety shown in the results sections below is not the overall factor of safety, but rather the one that satisfies the permissible probability of flooding, provided it is higher than the schematization factor. This is because the partial factors ${\gamma }_{n}$ and ${\gamma }_{d}$ have already been applied in the analysis.

## Effect of Installation of Displacement Piles (Case II)

**Fig. 2:**
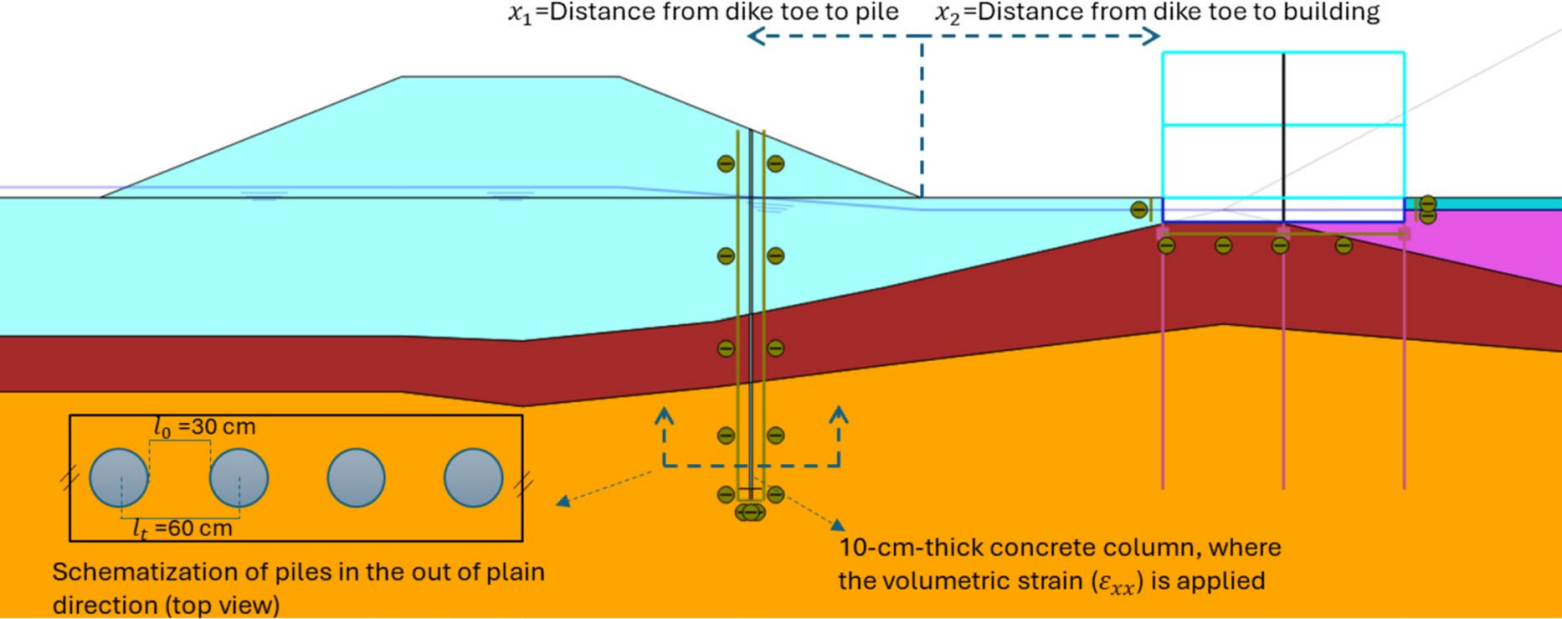
FEM model for examining the installation effect

The same geometry, soil properties, and hydraulic conditions as in the Bergambacht case are used to investigate the installation effects of displacement piles. It is not possible to directly simulate pile installation in the finite element method. Therefore, for displacement piles, an approach is utilized in which a lateral volumetric strain is applied to the soil. After reaching the condition of the existing dike and building, a thin concrete column in the ground is activated (Fig. 2), and then a volumetric strain in the lateral direction ${(\varepsilon }_{xx})$ is applied to push the surrounding soil outward. This stage is conducted under undrained soil conditions to assess the immediate (short-term) effects of soil displacement. The value of the volumetric strain depends on the diameter and spacing of the piles. For a plane strain model an equivalent diameter per unit length should be determined to calculate the appropriate volumetric strain to be applied. For a discontinuous wall, the equivalent stiffnesses per linear meter are determined by dividing the stiffnesses per linear meter of a solid wall with a correction factor ${f}_{opening}$. This factor is hereinafter referred to as 'opening factor' and it is given by:

3 $${f}_{opening}=\frac{{l}_{t}}{{l}_{t}-{l}_{0}}$$

where ${l}_{t}$ is the centre-to-centre distance between wall sections and ${l}_{0}$ is the length of the open part.

The same factor can be used to determine the equivalent diameter of a pile wall. However, for a pile wall that consists of circular piles, The ratio of the area of a circle inscribed in a square to the area of the square is π/4. The opening factor should be divided by this factor in the case of circular pile walls to match the volume of the piles.

For a pile diameter *D* = 30 cm and centre-to-centre spacing ${l}_{t}$=60 cm, the opening factor and equivalent diameter are given by:

4 $${f}_{\text{opening}}=\frac{{l}_{t}}{{l}_{t}-{l}_{0}}*\frac{4}{\pi }=\frac{60}{60-30}*\frac{4}{\pi }=2.55$$

5 $${D}_{\text{eq}}=\frac{D}{{f}_{\text{opening}}}=\frac{30}{2.55}=11.76 \text{cm}$$

Since the concrete column width in the model is set to *d* = 10 cm, and the required lateral soil displacement is 11.76 cm, then the prescribed volumetric strain is given by:

6 $${\varepsilon }_{\text{xx}}=\frac{{D}_{eq}}{d}*100=117.6\%$$

Buildings constructed near river dikes in the Netherlands are typically founded on pile foundations due to the presence of very soft subsurface soils. These piles are designed to reach the deeper Pleistocene sand layer, ensuring sufficient bearing capacity and acceptable levels of settlement. In the model, plate elements are used to simulate the walls and basement of a 2-storey-building with a height of 6 m, while embedded beam elements simulate the piles. The piles are 15 m long and the properties of the structural elements of the building are shown in Table 6 and Table 7.

**Table 6:** Material properties of the building’s plates

Parameter	Name	Building plate	Basement plate	Unit
Material type	–	Elastic	Elastic	–
Weight	*w*	10	20	kN/m/m
Isotropic	–	Yes	Yes	
Axial stiffness1	*EA*_*1*_	${10}^{6}$	${10}^{6}$	kN/m
Bending stiffness	*EI*	${13*10}^{3}$	${13.10}^{3}$	kNm^2^/m
Poisson ratio	*ν*	0	0	–

**Table 7:** Material properties of the building’s piles (embedded beams)

Parameter	Name	Pile	Unit
Material type	–	Elastic	–
Unit weight	γ	7	kN/m^3^
Pile spacing	*L*_*spacing*_	3	m
Cross section type	–	Predefined	–
Pre-defined cross section type	–	Solid circular beam	–
Diameter	–	0.9	m
Stiffness	*E*	$10*{10}^{6}$	kN/m^2^
Axial skin resistance	*Axial skin resistance*	Layer dependant	–
	*T*_*max*_	500	kN/m
Lateral resistance	–	Unlimited	
Base resistance	*F*_*max*_	500	kN

Both the effect of pile diameter and distance from the building are studied by varying the equivalent diameter between 10 and 40 cm (${\varepsilon }_{xx}$ between 100 and 400%), and the pile location on the slope and crest of the dike. In addition, the distance between the building and the toe of the dike is varied between 1 and 25 m.

## Results

While geotechnical factors (like slope stability, soil strength, and groundwater flow) are the core of dike design, non-geotechnical factors can significantly influence the choice of the location of the pile wall. Equipment access, environmental protection, the location of roads and traffic are among many other factors that may influence the chosen location of reinforcement. However, here we focus only on the factor of safety for macro stability (Ultimate Limit State) and deformations (Serviceability Limit State). Thereby the optimal location of the wall can be found by varying the location of the pile and calculating the factor of safety and deformations at the crest and toes of the dike.

The following subsections present the resulting factors of safety and deformations based on the pile location in the dike (Sect. 5.1), and the horizontal displacement in the building caused by the installation of displacement piles (Sect. 5.2).

### Macro Stability and Deformations

**Fig. 3:**
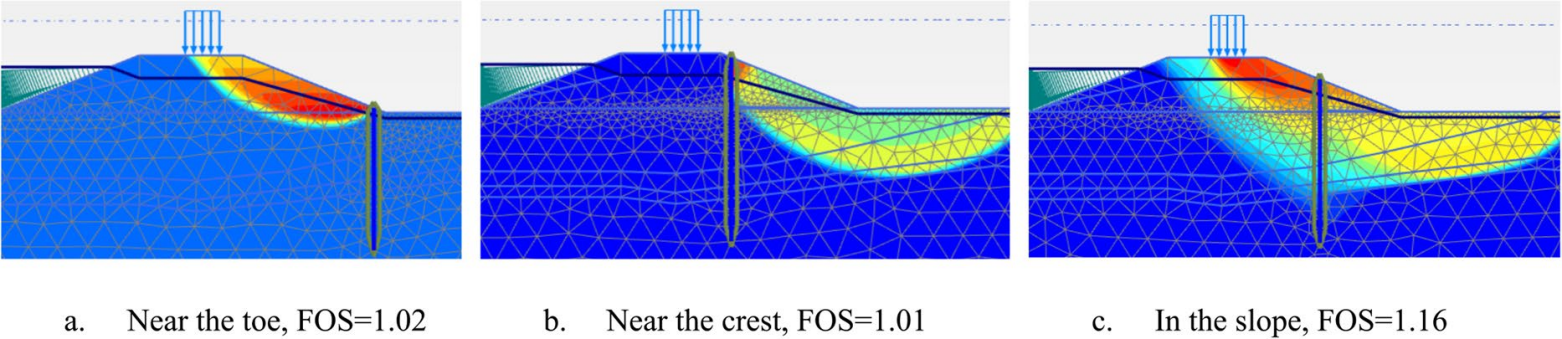
Failure mechanisms of the inner slope with different locations of the pile wall

The critical failure plane of the non-reinforced dike (commonly referred to as a “green dike”) under extreme water conditions is located on the inner slope and extends to the polder side. The green dike fails in Phase 4b when partial safety factors are applied, indicating a factor of safety less than one. To assess the effect of the pile wall position within the dike, its location was varied from 1 to 20 m from the toe, in 1-m intervals. Figure 3 shows the different failure mechanisms resulting from the FEM calculations, corresponding to various locations of the pile wall along the dike slope.

**Fig. 4:**
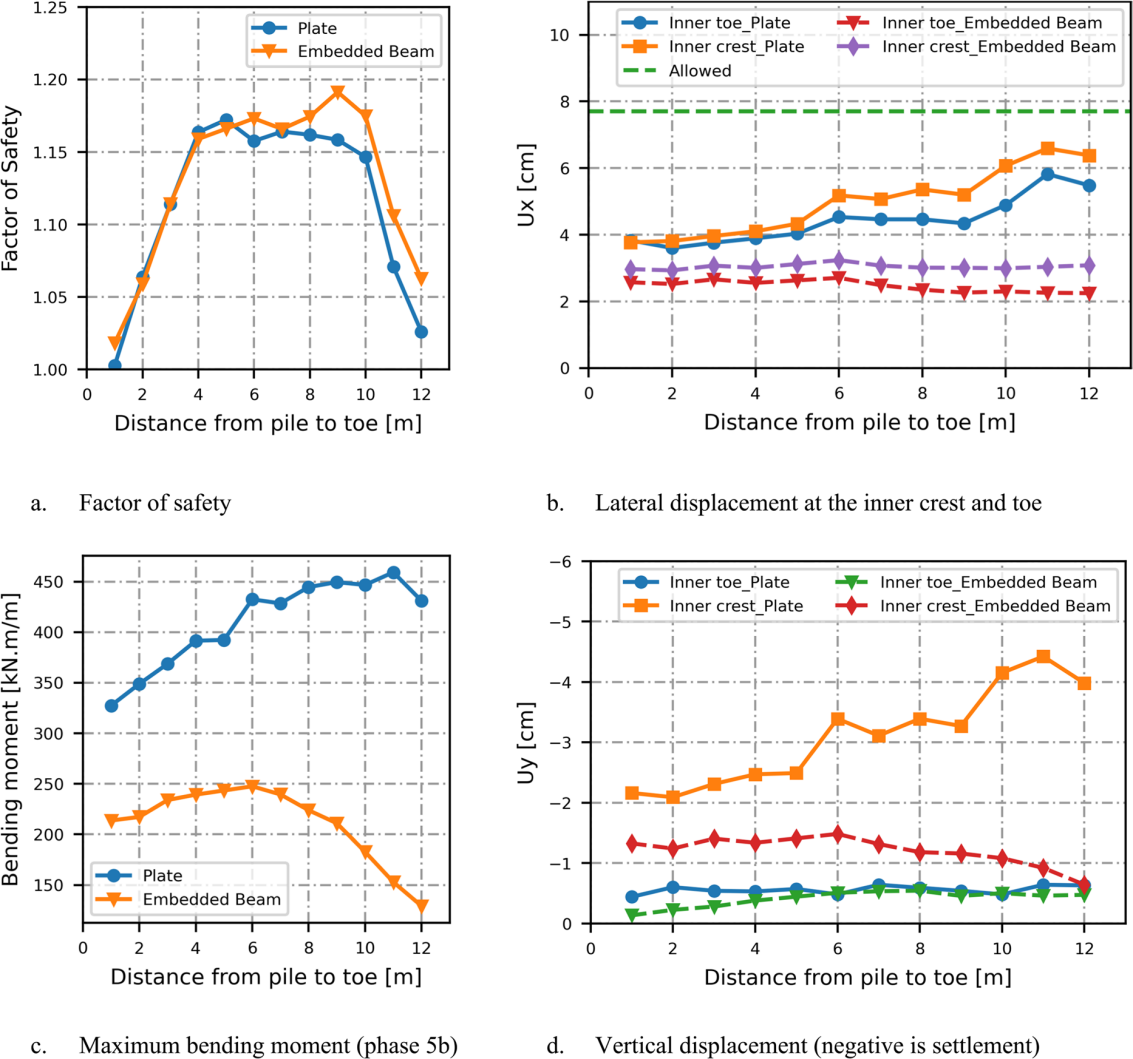
Effect of pile location in the slope on the factor of safety, displacements and bending moment

Placing the pile wall at the inner toe does not provide sufficient stability for the soil behind the wall. However, positioning the pile wall within the slope is effective for improving inner slope stability and is also more cost-effective, as it reduces the required pile length compared to placing it at the crest. As shown in Fig. 4a, the factor of safety increases with distance from the toe until a point is reached where the slope in front of the pile wall becomes unstable, causing the factor of safety to fall below the schematized partial factor of safety ${\gamma }_{b,GEO}$. In this case, the appropriate position for the pile wall is between 4 and 10 m from the toe.

The structural forces increase with greater distance of the piles from the toe, resulting in increased lateral displacement at the inner crest and toe, as shown in Fig. 4b for plate elements. While the factor of safety results are consistent between embedded beam and plate elements, the horizontal displacements at the inner crest and toe are lower for embedded beam elements compared to plate elements, particularly at larger distances of the pile wall from the toe, as illustrated in Fig. 4b.

The displacements should remain below 10 cm, with a partial factor of safety for displacements of 1.3 for the serviceability limit state (SLS). The allowable limit (7.7 cm) is not exceeded at the crest and toes of the dike, and the design satisfies the serviceability limit state.

The plate element exhibits greater bending moments than the embedded beams. The bending moment increases with the distance between the plate and the dike toe up to 11 m, after which it decreases. A similar trend is observed for the embedded beams; however, when the distance between the embedded beam and the dike toe exceeds six meters, the bending moment begins to decrease as shown in Fig. 4c. The vertical displacements at the inner toe remain low and appear to be only marginally influenced by the pile’s location within the dike, as illustrated in Fig. 4d. Settlements at the outer crest are higher for the plate elements compared to the embedded beams, particularly at greater distances between the pile and the dike toe, indicating lower resistance and a more conservative design.

When using a longitudinal structure in a dike, it is accepted under certain conditions that the slope behind the structure can shear. Such a shear is called a 'non-critical instability', because this shear does not have to directly influence the probability of flooding. The wall should be able to fulfil the retaining function independently in the event of sliding of the slope on the polder side or the water side. For bored piles, it should be ensured that they are safe against shearing and that soil does not flow into the space between the piles.

**Fig. 5:**
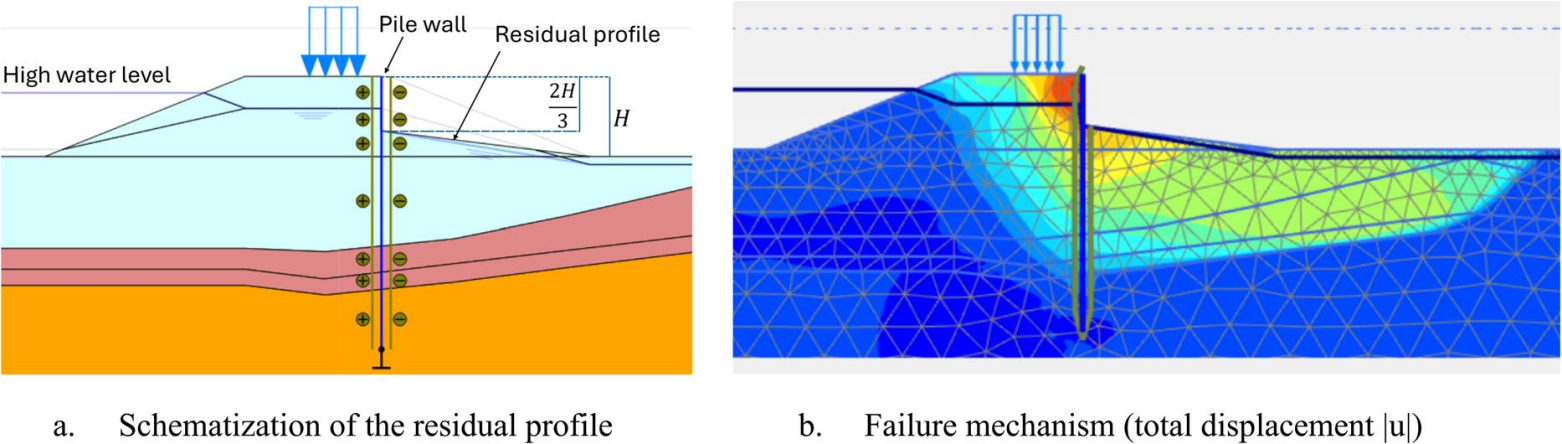
Schematization of the residual profile and failure mechanism

In this case, this instability occurs in the inner slope when the pile wall is at the crest during Phase 4b where the soil model for the soft soils is changed to the NGI-ADP-SHANSEP model and the partial factors of safety are applied to the shear strength properties of the soil. If such instability is identified during FEM checks, a schematized reduced residual profile must be applied after the installation of the longitudinal structure, in accordance with the guidelines. The schematized residual profile to be used set at one-third of the original height according to the (POVM 2020) is shown in Fig. 5a.

**Fig. 6:**
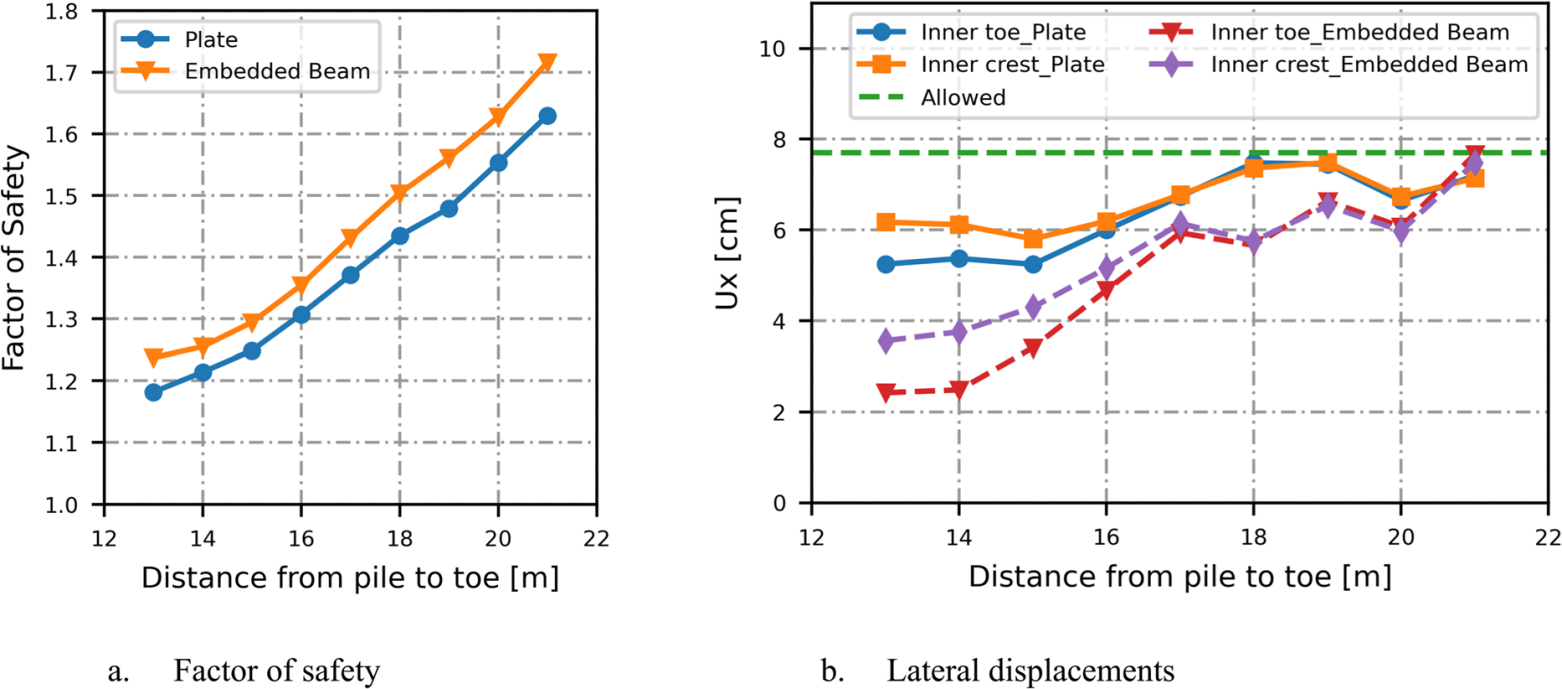
Effect of pile location in the crest on the factor of safety and lateral displacements

When the influence of the inner slope is excluded from the dike stability analysis, the slip surface shifts to the river side, and the factor of safety then depends on the ability of the pile wall to retain the soil behind it (Fig. 5b). As the soil pressure decreases with increasing the pile wall distance from the inner crest, the factor of safety correspondingly increases, as shown in Fig. 6a.

The serviceability limit state is still evaluated for the full profile as this concerns the situation without a ULS failure. The embedded beam resists the high pressures exerted by the soil and line load, and exhibits lower displacements compared to the plate element. When the pile wall is positioned further from the inner crest and beyond the line load (17.5 m from the toe) the displacements on the inner slope are minimally influenced by the structural element, and the results become comparable for both the embedded beam and plate elements (Fig. 6b).

When non-critical instability is not allowed, placing a pile wall within the inner slope of a dike, rather than at the toe or crest, provides the most effective balance between improving slope stability and minimizing structural demands on the pile. This position yields the highest factor of safety, reduces the required pile length (compared to crest placement), and keeps displacements within acceptable serviceability limits as shown in Fig. 4. While structural forces and lateral displacements increase with distance from the toe, these remain within design thresholds.

In cases where non-critical instability in the inner slope is allowed, positioning the pile wall at the crest results in a higher factor of safety, which increases with distance from the inner crest. However, this configuration also leads to greater displacements at both the inner crest and toe. Additionally, the design must ensure that soil does not flow into the gaps between piles if such non-critical instability occurs.

Modelling the pile wall with an embedded beam in the 2D plane strain model follows a different approach than modelling with the plate elements. The results of the factor of safety are in good agreement for both types of structural elements. However, in plate elements, the 3D effect is taken into account using a shell factor, which leads to lower stiffness and a more conservative design when compared to the embedded beam element, as the calculated horizontal displacements at the crest and inner toe of the dike were higher when using plate elements. This study used the default values for the interface stiffness factors of the embedded beam, which are calibrated for bored piles that are axially loaded with the surrounded soil modelled with the HS model. Using lower values than default for the interface stiffness factor leads to higher relative displacement between the pile wall and the soil. Therefore, it is recommended that the properties of the embedded beam elements are derived from measurement data from pile load test to verify the lateral resistance and the interface factors which highly depend on the installation method.

### Installation Effect

After determining the optimum location of the pile with respect to the factor of safety against macro-stability and deformations at the crest and toe of the dike, the impact of displacement pile installation on adjacent buildings is evaluated. This analysis is undrained, representing the immediate impact of pile installation. However, allowing pore pressure to dissipate (the long-term effect) only slightly influences the final deformations in the building.

The primary cause of damage in such situations is excessive horizontal displacement affecting the building’s foundation, potentially exceeding the serviceability limit state and, in some cases, causing structural damage. Eurocode 7 does not specify a fixed limit for allowable horizontal displacement, as it depends on various factors including the structure type and its sensitivity, soil conditions, foundation type, and local building codes. However, in practice, lateral displacements should generally remain below 1–2.5 cm for deep foundations, and below *H*/500 to *H*/1000 for buildings on shallow foundations, where *H* is the building height. Site-specific assessments are essential. In this case, a threshold of 2.5 cm is adopted as the permissible lateral displacement.

**Fig. 7:**
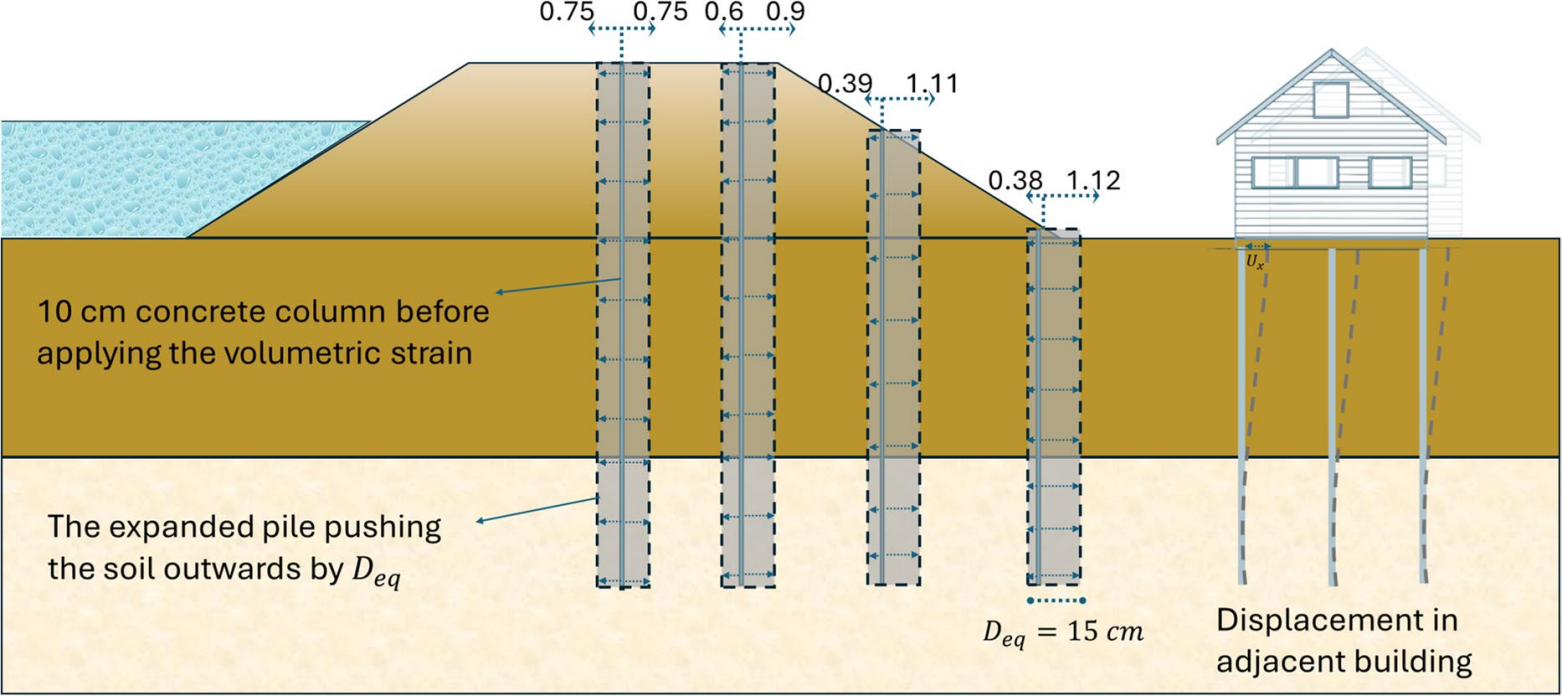
Lateral displacements in the concrete column elements (in cm) when applying volumetric strain ${(\varepsilon }_{xx})$ for a symmetric dike

Figure 7 illustrates how the concrete column in the model pushes the soil outward when volumetric strain is applied. For a horizontal ground surface with horizontal soil layers, the pile displaces the soil symmetrically on both sides during installation. However, this symmetry is disrupted when the ground surface and subsoil layers are inclined as shown in Fig. 7. This behaviour is explained by the stress-dependency of soil stiffness. According to the Hardening Soil model we have:

7 $${E}_{oed}={E}_{oed}^{ref}{\left(\frac{{\sigma }_{1}{\prime}}{{p}_{ref}}\right)}^{m}$$

For soft soil, a linear relationship is obtained when *m* = 1. Similarly, in the SSC model, there is a logarithmic relationship between changes in volumetric strain (*εᵥ*) and changes in mean effective stress (*p′*). This results in greater resistance in regions where the confining pressure is higher. Consequently, larger displacements are observed on the polder side when the pile is installed closer to the inner toe of the dike.

**Fig. 8:**
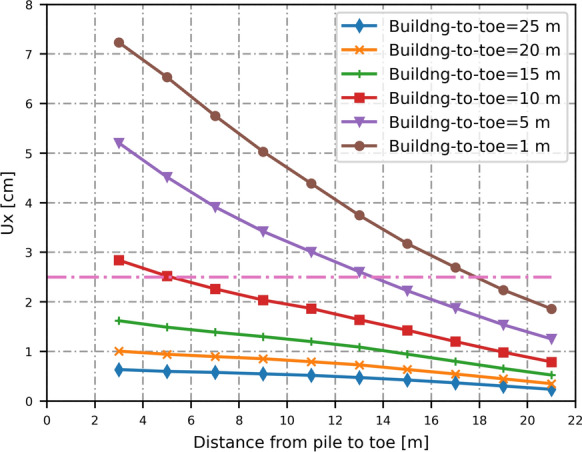
Maximum lateral displacement of building for pile wall of 30 cm diameter and 60 cm centre-to-centre spacing (*D*_*eq*_ = 11.76 cm)

For a pile wall with piles diameter of 30 cm and centre-to-centre spacing of 60 cm (*D*_*eq*_ = 11.76 cm), Fig. 8 shows the maximum horizontal displacement in the building foundation when the building is at a distance from the toe between 1 and 25 m. As can be seen, the horizontal displacement in the building depend on the location where piles are installed. The displacement decreases when increasing the distance from the pile to the dike toe. When the building is at a distance higher than 15 m, it is safe to place the piles near the toe. However, when the building is 10, 5 and 1 m away from the toe, it is only allowed to place the piles 5, 13 and 18 m away from the toe respectively.

**Fig. 9:**
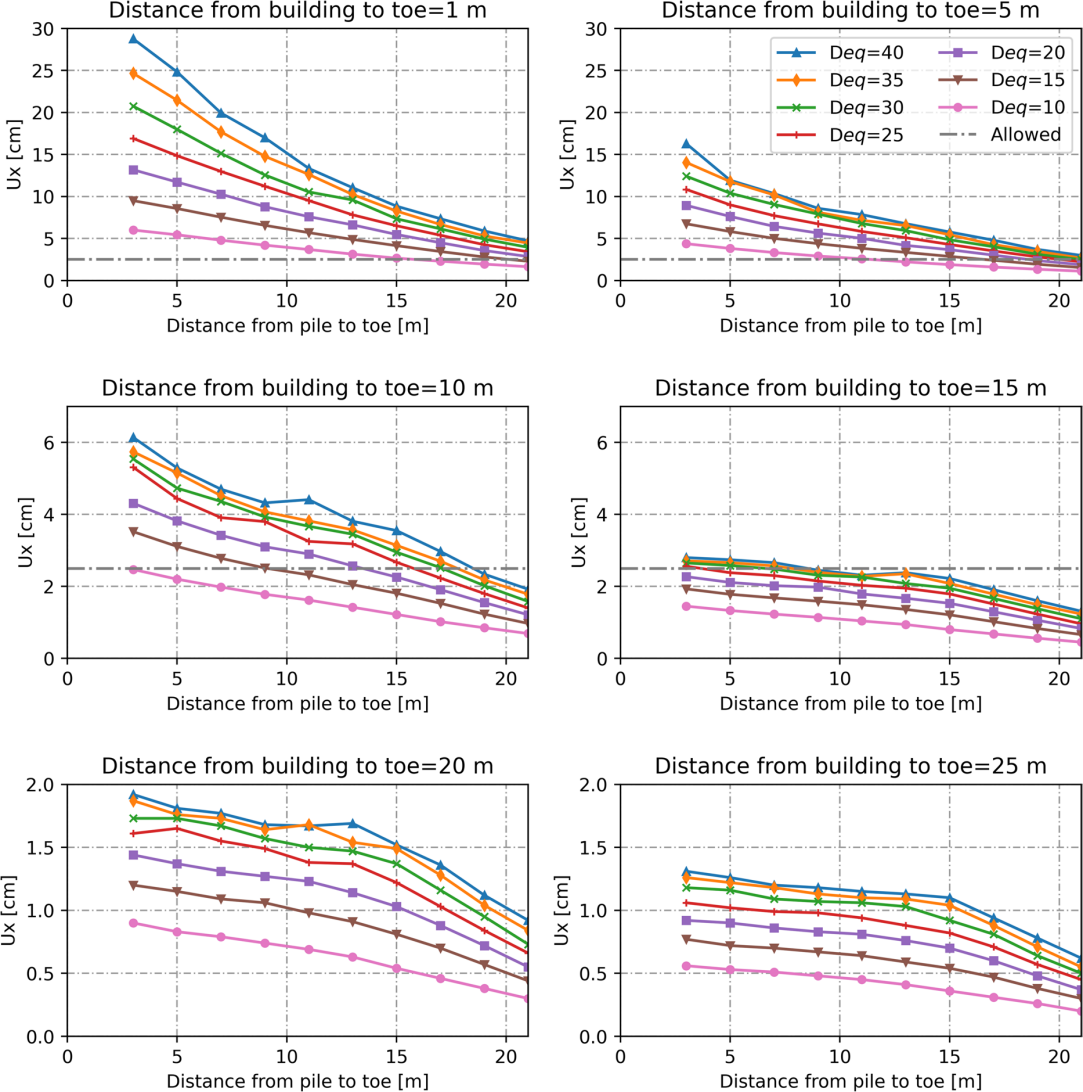
Lateral displacement in buildings due to installation of displacement piles with various equivalent diameters (in cm)

We can also examine the effect of different pile diameters and spacings on the building by varying the equivalent diameter of the pile wall. A larger diameter results in greater horizontal displacements in the building. As shown in Fig. 9, when the building is located 20 m or more from the pile, displacement piles with equivalent diameters greater than 40 cm can be installed near the inner toe. On the other hand, when the building is 15 m from the toe, and placing the pile within the inner slope is necessary, only pile walls with an equivalent diameter of up to 30 cm are suitable (e.g., *D* = *75* cm and *L*_*spacing*_ = 150 cm). When the building is just 5 m away from the dike, deformations become excessive if the pile is installed in the slope, and the use of displacement piles is not recommended in such cases.

The lateral deformations in the soil caused by the installation of displacement piles depend significantly on the density and stiffness of the surrounding soil. For example, installing piles in loose sand allows soil particles to rearrange and fill voids, leading to densification and lower stress transfer to adjacent structures compared to dense sand. A similar trend is observed in soft soil which is more compressible than stiff clay.

Since soil stiffness in advanced models is stress-dependent, multiple factors influence its determination. These include unit weight, over-consolidation ratio, and model-specific parameters such as λ*, κ*, and μ* in the SSC model, as well as *m, E₀, E₅₀*, and *Eₒₑ*_*d*_ in the HS model. This suggests that a sensitivity study would be complicated, considering all these parameters. However, rather than varying parameters individually in a sensitivity analysis, we could vary an overall soil property such as relative density or plasticity index or water content, from which a consistent set of model parameters are derived via correlations. This would simplify the sensitivity analysis, but still not easy.

To illustrate this influence, different soil types were used in the model to replace the dike material shown in Fig. 1. The horizontal displacement of the adjacent building was then calculated for a pile with an equivalent diameter (*D*_*eq*_) of 15 cm. The soil types range from stiff clay to light clay and very soft peat. Their properties are listed in Table 1.

**Fig. 10:**
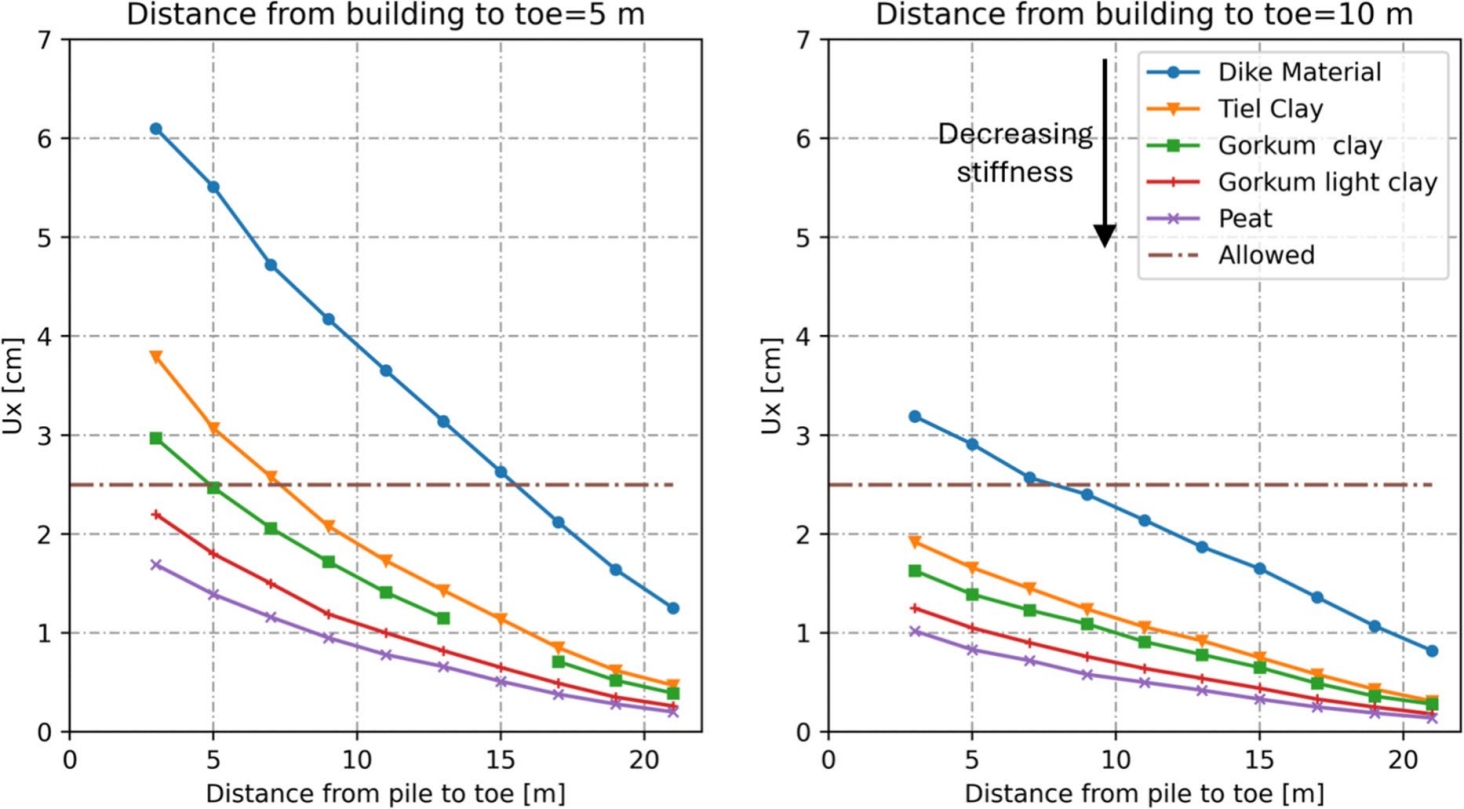
Effect of soil type and stiffness on the lateral displacements in the building due to installation of displacement piles with *D*_*eq*_ = 15 cm

Figure 10 shows that horizontal displacements in the building decrease with decreasing soil stiffness and unit weight, due to the lower stresses transmitted to its foundations.

Although this might suggest that installing piles in softer soils is safer when buildings are located closer to the toe, it is important to note that the model accounts only for horizontal displacements resulting from the pile pushing the soil outward. It does not consider deformations caused by heavy equipment during pile installation, nor the vibrations and potential pore pressure development associated with the driving technique. These additional effects could complicate the installation process in softer soils and potentially cause more damage than the model predicts. Moreover, softer soils have lower shear strength and therefore require pile walls with larger diameters, which as previously shown, leads to greater lateral displacements.

## Conclusion

This study employed the finite element method (FEM) using PLAXIS to analyse the effects of displacement pile walls on dike stability and adjacent buildings, using the Bergambacht dike as a representative case in the Netherlands. Structural components such as the pile wall and building foundation were simulated using embedded beam and plate elements, allowing for detailed evaluation of interactions between soil and structure under various loading and installation conditions.

The results demonstrate that placing the pile wall within the inner slope of the dike offers the most favourable balance between improving macro stability and maintaining serviceability limits. This configuration yields the highest factor of safety while controlling horizontal displacements at the crest and toe of the dike, making it the most effective location when non-critical instability is not allowed. Conversely, positioning the pile wall at the crest improves global stability if slope shearing is accepted as non-critical but leads to increased lateral displacements and higher structural demands. A comparison between plate elements and embedded beams revealed that while both provide similar trends in factor of safety, embedded beam elements resulted in lower horizontal displacements due to their higher stiffness representation. Plate elements, incorporating a shell reduction factor to account for 3D effects, tend to yield more conservative results with higher deformation values. Therefore, while either method can be used, careful calibration of model parameters is required to ensure accurate predictions.

The study also examined the installation effects of displacement piles, which were simulated by prescribing lateral volumetric strain to represent the pile pushing the soil outwards. The magnitude of the prescribed strain depends on the equivalent pile diameter, which itself is a function of pile spacing and actual diameter. This installation effect was found to induce significant horizontal displacements in nearby structures, particularly when piles are placed close to buildings or when larger diameters are used. For buildings located less than 15 m from the dike toe (for this case study), only smaller-diameter piles or greater installation distances could keep displacements within the acceptable threshold. In Bergambacht-Schoonhoven (BAS) dike, the piles were closer to some of the buildings than recommended in this study, which resulted in damage to these buildings.

Furthermore, soil type and stiffness were found to significantly influence the displacement behaviour during pile installation. While softer soils tend to reduce stress transfer to adjacent structures and thus show smaller horizontal displacements, they also pose challenges due to their lower shear strength and increased risk of deformation under equipment loads and vibration, factors not captured in the numerical model but relevant for real-world construction.

## List of symbols

${{S}_{u}}^{A}$: Active undrained shear strength
${{S}_{u}}^{\text{DSS}}$: Undrained shear strength in direct simple shear
${{S}_{u}}^{P}$: Passive undrained shear strength
${{\gamma }_{f}}^{C}$: Peak shear strain in compression
${{\gamma }_{f}}^{E}$: Peak shear strain in extension
${{\gamma }_{f}}^{\text{DSS}}$: Peak shear strain in direct simple shear
*G*: Shear modulus
${\tau }_{0}$: Mobilized shear strength
$\nu$: Drained poisson ration
${\nu }_{u}$: Undrained poisson ration
${S}_{u}$: Undrained shear strength
${\sigma }_{1}{\prime}$: The largest effective principal stress
${\sigma }_{1,\text{max}}{\prime}$: The current pre-consolidation stress
$\alpha$: Normalized soil parameter for SHANSEP
*m*: Normalized soil parameter for SHANSEP
*SSC*: Soft soil creep
*HS*: Hardening soil
*NGI-ADP*: Norwegian geotechnical institute's active-direct shear-passive (model)
*SHANSEP*: Stress history and normalized soil engineering properties
*FEM*: Finite element method
*BAS*: Bergambacht–schoonhoven
*KIS*: Kinderdijk–schoonhovense veer
*PL1*: Average water level
*PL2*: Extreme water level
*NAP*: Normaal Amsterdams Pei (normal amsterdam level)
*PL3*: Pressure in aquifer at daily conditions
*PL4*: Pressure in aquifer at extreme conditions
${\gamma }_{\text{sat}}$: Saturated unit weight
${\lambda }^{*}$: Modified compression index
${\kappa }^{*}$: Modified swelling index
${\mu }^{*}$: Modified creep index
$${\nu }_{\text{ur}}$$: Poisson ratio
$${c}{\prime}$$: Cohesion
$${\phi }{\prime}$$: Friction angle
$${\psi }{\prime}$$: Dilatancy angle
${R}_{\text{int}}$: Interface reduction factor
*K*_*0*_^*nc*^: At-rest earth pressure coefficient for normally consolidated soil
*POP*: Pre-Overburden pressure
${E}_{50}$: Secant stiffness (triaxial)
${E}_{\text{Oed}}$: Tangent stiffness (oedometer)
${E}_{\text{ur}}$: Unloading/reloading stiffness
*m*: Stress dependency power
*R*_*f*_: Failure ratio
*FOS*: Factor of Safety
*ε**ₓₓ*: Horizontal volumetric strain
*EI*: Bending stiffness
*EA*: Axial stiffness
*D*: Pile diameter
*S*: Shell factor
$${f}_{\text{opening}}$$: Opening factor
${l}_{t}$: Centre-to-centre distance between wall sections
${l}_{0}$: Length of the open part between wall sections
${D}_{\text{eq}}$: Equivalent diameter for non-continuous walls
*d*: Width of concrete column in the model
*T*_max_: Maximum axil skin resistance
*F*_max_: Maximum base resistance
$${\gamma }_{n}$$: Model factor
$${\gamma }_{d}$$: Damage factor
$${\gamma }_{b,\text{GEO}}$$: Schematization factor
*H*: Building height
*U*_*x*_: Lateral displacement
*U*_*y*_: Vertical displacement
${p}_{\text{ref}}$: Reference mean effective stress
*p**′*: Mean effective stress
*εᵥ*: Volumetric strain
